# Air Pollution Exposure in Relation to the Commute to School: A Bradford UK Case Study

**DOI:** 10.3390/ijerph13111064

**Published:** 2016-10-29

**Authors:** Kim N. Dirks, Judith Y. T. Wang, Amirul Khan, Christopher Rushton

**Affiliations:** 1 Faculty of Medical and Health Sciences, School of Population Health, University of Auckland, Auckland 1142, New Zealand; 2 School of Civil Engineering, University of Leeds, Leeds LS2 9JT, UK; j.y.t.wang@leeds.ac.uk (J.Y.T.W.); a.khan@leeds.ac.uk (A.K.); 3 Institute for Transport Studies, University of Leeds, Leeds LS2 9JT, UK; phy4cer@leeds.ac.uk

**Keywords:** pedestrian, traffic, ultrafine particles, school, children, exposure

## Abstract

Walking School Buses (WSBs) provide a safe alternative to being driven to school. Children benefit from the contribution the exercise provides towards their daily exercise target, it gives children practical experience with respect to road safety and it helps to relieve traffic congestion around the entrance to their school. Walking routes are designed largely based in road safety considerations, catchment need and the availability of parent support. However, little attention is given to the air pollution exposure experienced by children during their journey to school, despite the commuting microenvironment being an important contributor to a child’s daily air pollution exposure. This study aims to quantify the air pollution exposure experienced by children walking to school and those being driven by car. A school was chosen in Bradford, UK. Three adult participants carried out the journey to and from school, each carrying a P-Trak ultrafine particle (UFP) count monitor. One participant travelled the journey to school by car while the other two walked, each on opposite sides of the road for the majority of the journey. Data collection was carried out over a period of two weeks, for a total of five journeys to school in the morning and five on the way home at the end of the school day. Results of the study suggest that car commuters experience lower levels of air pollution dose due to lower exposure and reduced commute times. The largest reductions in exposure for pedestrians can be achieved by avoiding close proximity to traffic queuing up at intersections, and, where possible, walking on the side of the road opposite the traffic, especially during the morning commuting period. Major intersections should also be avoided as they were associated with peak exposures. Steps to ensure that the phasing of lights is optimised to minimise pedestrian waiting time would also help reduce exposure. If possible, busy roads should be avoided altogether. By the careful design of WSB routes, taking into account air pollution, children will be able to experience the benefits that walking to school brings while minimizing their air pollution exposure during their commute to and from school.

## 1. Introduction

In societies where many children are driven to school every day, a Walking School Bus (WSB), a school-based initiative in which children walk to school in a group supervised by adults and following established routes, is an attractive alternative. WSBs help to reduce car congestion near the school entrance (thereby reducing the accident risk in the immediate vicinity of the school) and provide an opportunity for children to learn about road traffic safety by experiencing the road environment under adult supervision [1]. WSBs also contribute towards recommended daily exercise targets for children; in the UK, this is set at 60 min per day [2]. Travel time may also be reduced by walking if there is severe road traffic congestion along the route and parking in the vicinity of the school is problematic at key times of the day. Based on the UK National Travel Survey, walking is a significant mode of transport for primary school children: 46% of five- to 10-year-olds walk to school, and 57% of seven- to 13-year-olds that walk to school are usually accompanied by an adult [3].

One of the main barriers to walking identified by parents who drive their children is road safety. Traffic danger is considered the most commonly mentioned reason (58%) for adults to accompany school trips [3]. This fear is not without basis—pedestrian accidents are a main cause of death amongst children in developing countries, with the leading cause amongst school-aged children being the journey to school. A UK study has suggested that 50% of injuries in school-aged children result from collisions between cars and pedestrians or cyclists [4]. More recent statistics (complied by the research group Road Safety Analysis and Axa Car Insurance) have shown that between 2006 and 2011, there were over 550,000 vehicle collisions around schools (equivalent to six collisions per school per year on average across the UK), and there were over 85,000 child injuries on roads within a 500 m radius of the school (cited in [5]). Compared with walking alone, WSBs help to reduce the risk of traffic-related accidents by providing adult supervision, and help to support children in their preference for walking to school [6]. They also help break the cycle of car dependency [7], with the potential to benefit society through improved air quality and also reduced road traffic noise for the local residents.

The WSB idea was first trialed at Wheatfields Junior School in St Albans, UK, in 1998 [8], and was subsequently adopted in several other developed nations around the world including in Canada, the USA, Australia and New Zealand [8]. As of 2005, WSBs existed in over 150 school across the UK and in 100 schools in New Zealand [8].

Despite its popularity, the uptake of the WSB is not spatially homogenous. Analysis of the distribution of WSBs suggests that they are significantly more common in wealthy neighborhoods where childhood pedestrian injury rates are typically low but where parental engagement is likely to be the highest [9]. The provision of WSBs is currently based largely on buy-in from the school in question, catchment need and the availability of “bus drivers”, i.e., parents willing to contribute to the initiative on a regular basis and keep the bus active. The specific route the bus follows takes into account traffic safety (particularly at road crossings) as well as travel time.

One safety consideration that is rarely mentioned is the exposure to air pollution during the commute to school. For many people, the commute to and from work or school is a significant contributor to an individual’s daily air pollution dose as the space in the immediate vicinity of roads tends to be a high-exposure microenvironment for traffic air pollution. Research carried out investigating air pollution exposure associated with different modes of commuting suggests that exposure is slightly lower for pedestrians than for car commuters due to their increased separation from the main line of traffic [10]. However, pedestrians are at greater risk of exposure to short-term peaks due to the lack of a physical barrier between the source (the exhaust pipe) and their respiratory system. Also, when the breathing rate (or the minute ventilation) of a pedestrian is taken into account, it has been found that the amount of pollution that is inspired is higher for pedestrians compared with sedentary car commuters [11]. Depending on the level of traffic congestion, the amount of time spent in the commuting microenvironment may be higher for pedestrians compared with car commuters, further contributing to the air pollution dose associated with the commute [11].

To the best of our knowledge, no study has investigated route choice in the journey to school for pedestrians and the implications for pollution exposure for children. Also, little is known about the relative exposure to air pollution experienced by children walking to school relative to those who travel to school by car. This paper investigates the impact of mode and route choice (namely the side of the road travelled on) on air pollution exposure in the journey to school for a school located along a road with high levels of traffic, based on air quality data collected during a two-week field campaign of a hypothetical WSB route, with the goal of identifying ways in which exposure can be minimised.

## 2. Materials and Methods

### 2.1. Site Selection

The study site is located in the City of Bradford, 14 km to the west of Leeds in the UK. Bradford experiences a marine climate with an average annual precipitation of 872 mm. Data collection was carried out in November during which time the daily average maximum and minimum temperatures are 9.3 °C and 3.9 °C, respectively, with an average total monthly precipitation of 86.7 mm [12]. Air pollution levels tend to be high during the winter due to reduced thermal convection but washout of pollutants due to rainfall is also common.

The school chosen is Wycliffe Church of England Primary, in Shipley on Saltaire Road, located to the northwest of the city centre. The school has a roll of about 226 pupils aged from nine to 13 years. The road immediately outside of the school, Saltaire Road, carries approximately 1600 ± 200 vehicles per hour from 8–9 a.m. and 1500 ± 100 from 3–4 p.m. at the end of the school day (based on traffic counts collected during the field campaign). This school was chosen in part because of its location on a busy arterial road. It has been estimated that 51% of the children enrolled in the school walk to school, 43% travel to school by car, 4% take the bus and the remainder travel by bicycle or take the train (less than 1% each) based on a survey carried out by the Born in Bradford team (unpublished).

### 2.2. Route Description and Field Trials

**Figure 1:**
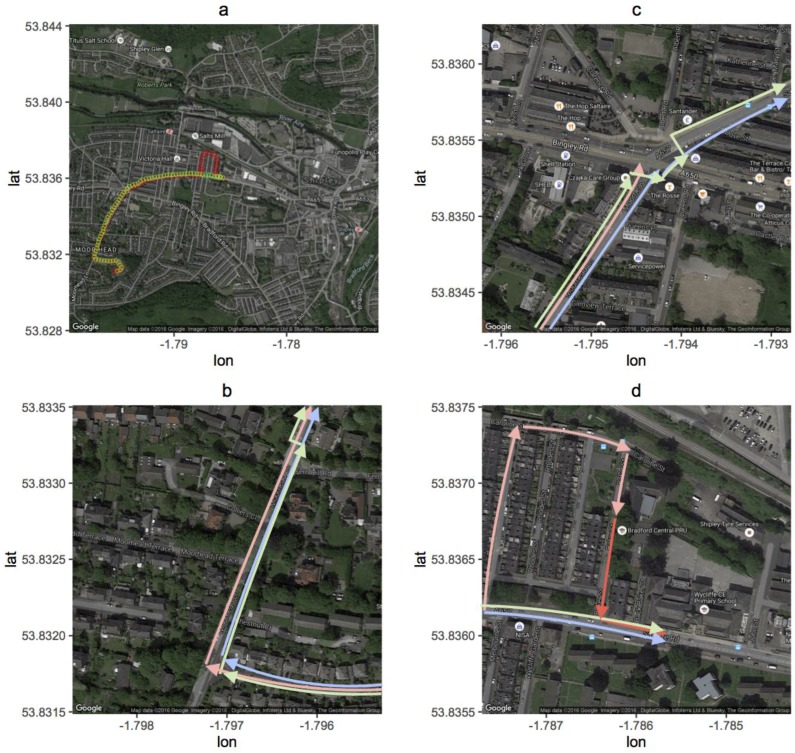
Map depicting the route to school in the morning. (**a**) The journey to school begins in the bottom left-hand corner (the house) and finishes in the top right-hand corner (the school). The green markers represent the route for the walkers while the red markers are those for the car commuter. The detour at the end of the journey for the car commuter is for parking the car; (**b**) The beginning of the route (red is the car, green is the North Walker and blue is the South Walker; (**c**) The intersection. (**d**) The final segment of the journey close to the school.

The field trials involved three commuters: one travelling to school by car and two travelling on foot, along the same road but, for the majority of the journey, travelling on opposite sides of the road. The walking route is approximately 1.4 km in length (see Figure 1a). It consists of short segment (200 m) along a quiet road (Roundwood Road), a 0.5 km segment along which the traffic heavily dominated in one direction (Morrhead Lane), a major intersection, and then another segment along which traffic is heavy on both directions (Saltaire Road). The route starts from the end of a cul-de-sac on Roundwood Road, a road consisting of very low traffic flows (0–1 vehicles passing in the 200 m, 5 min walking journey) (see Figure 1b). Along this road, for the purpose of this study, the two pedestrians walked on opposite sides of the road. The pedestrians then turned onto Moorhead Lane, initially, due to a lack of opportunity to safely cross the road, travelling on the same side of the road, until they reached a pedestrian crossing partway along the road. At this point, one of the pedestrians (Walker North) crossed the road onto the western side of the road, and travelled along Moorhead Lane, aligned as much as possible with the other walker (Walker South) who remained on the east side of the road. Both pedestrians then travelled approximately 8 min (0.5 km) down to the main intersection with Saltaire Road. The crossing of Saltaire Road, crossed together by the two pedestrians, required navigating a number of signalised pedestrian crossings with a “Lollipop Man” responsible for helping children to cross the intersection safely (see Figure 1c). Once this had been navigated, (with the North Walker traversing one addition road crossing to reach the north side of Saltaire Road), the rest of the journey (0.7 km taking approximately 10 min) consisted of a commute along Saltaire Road until reaching the school, with the Walker North travelling on the north side of the road and the Walker South travelling on the south (see Figure 1d). The journey was considered complete when the Walker South had crossed the pedestrian crossing over to the north side of the road to the front gates of the school. As much as possible, the pace of the walkers was set at 4.2 km/h, a pace assumed to be typical of a 10-year-old child walking to school. In the afternoon, the walk home consisted essentially of the same route home as in the morning but travelled in reverse.

The car commuter drove the same route but turned off Saltaire Road before the school. Once a carparking space has been secured (this changed from day to day depending on availability), the commuter travelled the rest of the journey to the front gate of the school on foot, with the pedestrian path taken changing somewhat depending on where the parent was able to park on the day. The journey in the afternoon was the reverse of that travelled in the morning. For all journeys, the car’s ventilation system was set to “new air” (“recirculate” turned off), with the car completely ventilated at the beginning of the commute so that the air pollution levels inside the car started at the same level, as that outside of the vehicle and experienced by the walkers.

### 2.3. Air Quality Data

Each participant was equipped with an ultrafine particle counter (P-Trak) logging exposures at 10 s resolution throughout the period of the commute. The device was carried in the hand by the walkers with the inlet of the device facing forward. For the car commuter, the monitor was placed on the front seat with the sensor exposed to the in-vehicle air. Commuting in the morning began at 8:20 a.m. to ensure arrival at 8:40 a.m. in time for the commencement of school at 8:45 a.m. Travel home at the end of the day started at 3:20 p.m. allowing time for packing up for school that ends at 3 p.m. Note that the start and commute times for the pedestrians were fixed to ensure travel on the same road segments at the same time each day and that all pedestrian commutes were 20 min.

Data were collected over a period of two weeks and consisted of five days of data collection during morning and in the afternoon, but not always on the same day. Rainy days were excluded as the equipment is not able to be operated if the conditions are wet.

### 2.4. Statistical Analysis

Statistical analysis was conducted using IBM SPSS Statistics Version 22 software (IBM SPSS, Chicago, IL, USA) and consisted mainly of unpaired and paired *t*-tests based on comparisons between commuters, road segments or times of the day.

## 3. Results

Table 1 presents the descriptive statistics for the study. The journey consisted of 120 periods of 10 s or a total of 20 min. For the car commuter, the travel time varied from day to day, depending on the traffic flow and the availability of a suitable place to park, but the journey began at a fixed time to match that of the pedestrians. The time taken for the journey by car was consistently shorter than for the pedestrians, ranging from 9–18 min.

**Figure 2:**
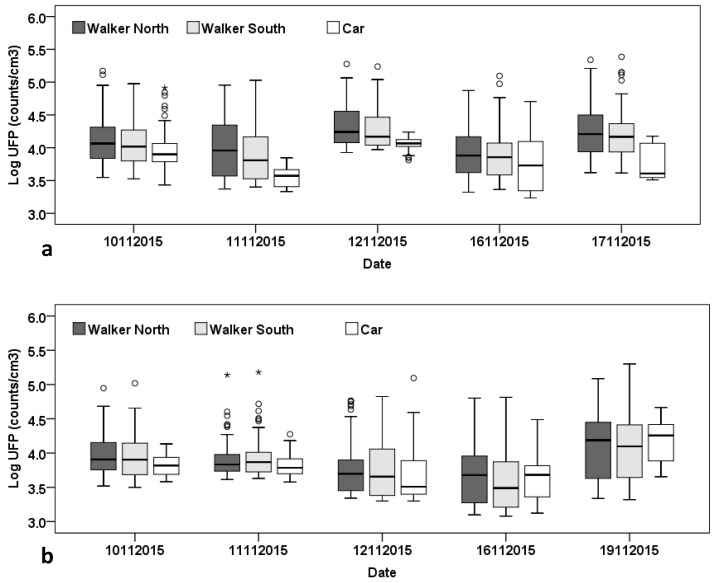
Box plots of the log-transformed UFP concentrations for each commute in the field campaign by commuter; (**a**) Morning commutes; (**b**) Afternoon commutes.

Figure 2 shows the commute average ultrafine particle (UFP) concentrations for both the morning and afternoon commutes over the period of observation for each of the modes and walking routes. Note that the average exposures vary from day to day (by a factor of two or three or so) both in the morning and in the afternoon for both of the walkers as well as the car commuter. This is partly due to the variability in meteorological conditions from day to day and also day-to-day variability in the traffic flows. Levels are generally lower in the afternoon compared with the morning, except for the afternoon of the 19 November, when levels were high for all commuters, due at least in part to the calm wind conditions experienced during that period.

**Figure 3:**
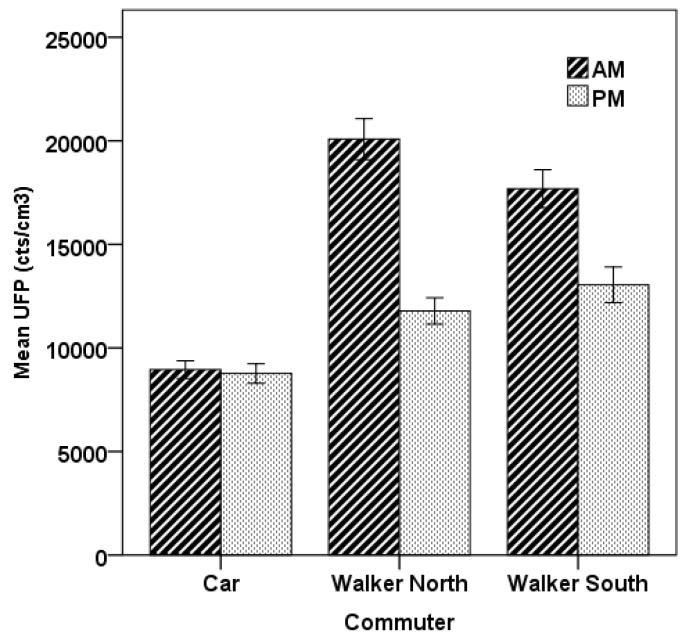
Route average UFP comparison between modes and routes. The error bars are the standard errors of the mean (SEM) for the five commutes in each category.

Figure 3 compares the commute mean averages for each of the two modes and three routes for both the morning and afternoon commutes. It shows that the exposures experienced by the walkers are significantly higher than for the car commuter for both the morning (North Walker t = 3.52, *p* = 0.02 and South Walker t = 3.54, *p* = 0.024) and the afternoon (North Walker t = 3.98, *p* = 0.016 and South Walker t = 3.31, *p* = 0.03) based on paired *t*-tests, with the differences most pronounced in the morning. No significant differences were found between the route average exposures for the North Walker North and South Walker in either the morning (t = 2.77, *p* = 0.05) or in the afternoon (t = 1.61, *p* = 0.18), nor for the North Walker between morning and afternoon (t = 1.95, *p* = 0.087) or the South Walker between morning and afternoon (t = 1.07, *p* = 0.31). Further, no significant difference was found between the morning and afternoon commutes for the car commuter (t = 0.113, *p* = 0.91).

**Figure 4:**
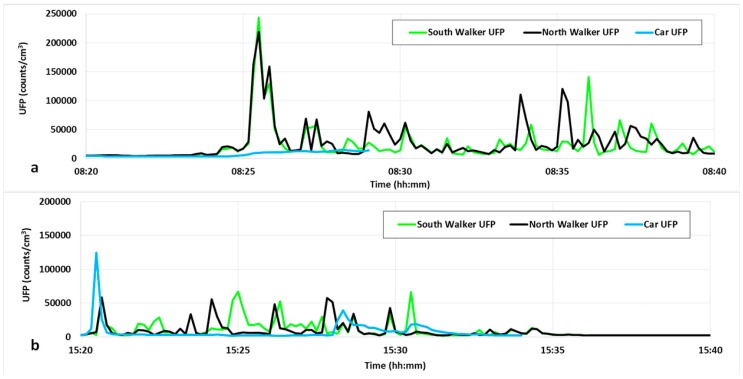
Example of UFP exposure for a commute (**a**) 17 November 2015 morning and (**b**) 12 November 2015 afternoon for the two walking routes and the car commuter. Note the peak in concentration that occurs shortly after 8:25 a.m. for the pedestrians when they reach a partially enclosed stretch of road and the shorter duration of the commutes for the car commuter.

Figure 4 shows two examples of the time series of UFP exposure (one for the morning commute and one for the afternoon commute) for both of the walkers and also the car commuter. Note that for both of the walkers, the concentrations remain low for the time spent along Roundwood Road where the traffic flows are very light. In the morning, a large peak is observed soon after the walkers reach Moorhead Lane, where the traffic is heavy and the road is enclosed with a tall brick wall on one side (and a very narrow footpath) and tall overhanging trees on the other side, suggesting some trapping of air. After the North Walker crosses the road at the pedestrian crossing, the route requires the pedestrian to pass cars that are queued up for the major intersection. The small peaks from 8:28–8:30 a.m. for the North Walker are largely absent for the South Walker travelling on the side opposite to the queued traffic. Peaks are also observed for both walkers as they walk along the Saltaire Road segment. The car commuter experiences relatively low exposure throughout the journey, except near the end for the short walk to the school from where the car has been parked. In the return journey at the end of the school day, peaks are observed for both pedestrians along the length of Saltaire Road and through the major crossing. Some peaks are observed for the North Walker along the Moorhead Lane component while passing traffic queued for the intersection, though the peaks are less frequent and generally smaller than during the morning commute. Exposures for the pedestrians are relatively low for the remainder of the journey. For the car commuter, some modest peaks are observed while walking to the car and also while driving along Saltaire Road with levels very low beyond that point.

**Figure 5:**
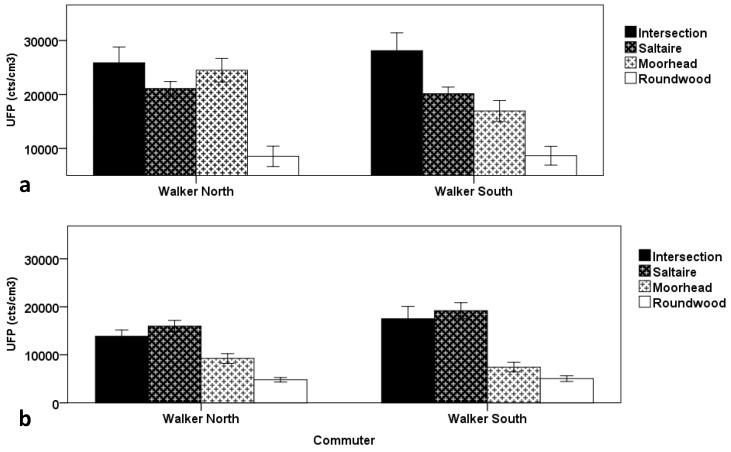
Mean UFP concentration by road segment for walkers: (**a**) Morning commute, (**b**) Afternoon commute. The error bars are the standard errors of the mean (SEM) for the five commutes in each category.

Figure 5 shows the commute mean UFP exposure split by time of day as well as by road segment, whether Roundwood, Moorhead, Saltaire, or the time spent passing through the intersection, focussing only on the two walkers. During the morning, the highest averages are experienced while in the intersection segment, either waiting to cross at the signalised pedestrian crossing or while crossing. During the afternoon, the levels experienced at the intersection are also high, though slightly lower than while travelling along the heavily congested Saltaire Road. High levels are also experience by the walker travelling on the north side of the road along Moorhead Lane, immediately adjacent to queued traffic entering the main intersection along the route. The Roundwood road segment, devoid of any significant traffic, consistently results in the lowest mean exposure amongst all of the road segments, both in the morning and in the afternoon.

**Figure 6:**
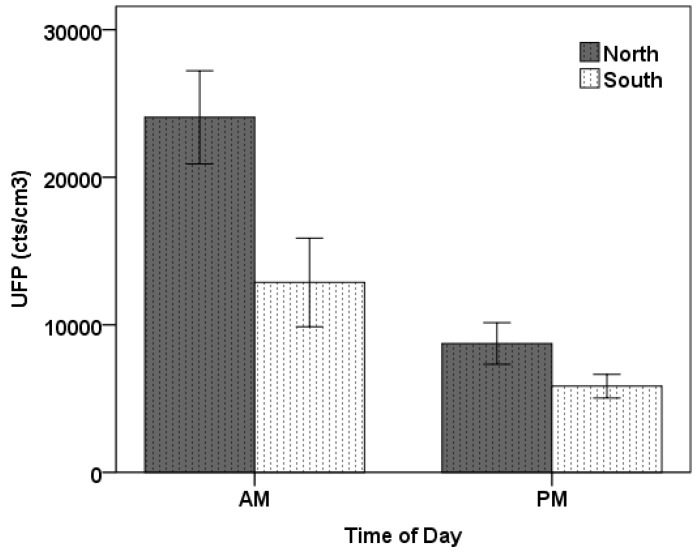
Mean UFP concentration for the Moorhead Road segment. The error bars are the standard errors of the mean (SEM) for the five commutes in each category.

For this particular route, most of the time, the walkers travelled on opposite sides of the road. However, for a short segment of the route, due to constraints with respect to safely crossing the road, the two commuters travelled essentially in tandem on the same side. Figure 6 compares the commute mean concentrations when isolating only the Moorhead Lane segment of the commute, separated into the segments where commuters travelled on opposite sides and on the same side for both the morning and afternoon commutes. This figure shows that the mean concentration is significantly higher (t = 4.12, *p* = 0.009) (by a factor of about two) when travelling on the side with the queued traffic (South) compared to the side opposite (North) for the morning commute based on a paired t-test. There is no significant difference for the afternoon commute (t = 2.119, *p* = 0.10).

## 4. Discussion

Exposures for the pedestrian commutes to and from school varied significantly depending on the environment in which they travelled. Traffic congestion clearly played a part, with some of the highest concentrations observed occurring in the vicinity of the major intersection experienced halfway along the route. Efforts to phase the lights to optimise pedestrian traffic and reduce waiting times, especially at school commute times and key intersections near schools, would help to reduce the air pollution exposure experienced by children while walking on their journey to school. Pedestrian travel immediately adjacent to queued traffic was associated with higher mean exposure than for those walking on the opposite side of the road alongside free-flow traffic. This is consistent with the literature, suggesting that increases in separation between the road and the commuter reduce exposure for active mode commuters [10]. Routes avoiding the queued side of the road are preferable in terms of exposure minimisation.

Despite the relative heavy congestion on the main road outside of the school, the commute times for the walkers were consistently longer than for the car commuters due to the relatively long segment of road consisting of light traffic flows at the house end of the commute. Air pollution exposure experienced by the walkers was also higher, especially on the more congested segments of the route. With air pollution exposure and travel time both contributing to air pollution “dose” (mean concentration times exposure time), the dose is clearly higher for pedestrian commuters than for car commuters for this journey to school. At some very high level of congestion, one could expect that the travel time of the car commuter would become sufficiently long that the reduced travel time of pedestrians would eventually compensate for the increased average exposure.

This study is limited by the fact that the dataset consists of only five mornings and five afternoons. This is in part due to the fact that data collection was not possible when it was raining and a wet season was chosen for the study. However, the statistical power was improved by ensuring that comparisons between modes and sides of the road for the pedestrians could be made using paired *t*-tests, thereby controlling for the day-to-day variability in concentrations due to the meteorology and traffic conditions. The fact that significant differences were found suggests that the dataset was adequate for the purpose. The study was conducted over a short period at one time of the year. The extent to which the results would vary between seasons remains to be investigated.

The study was also limited in that only the side of the road was considered in terms of “route choice”. In many situations, as well as the side of the road, there are also options to travel through areas of green space and along routes with significantly reduced traffic flows. If this study had considered such routes, there is no doubt that the exposures would have been significantly reduced. The study design is therefore limited to situations in which the “bus route” and its stops are constrained to the main road.

It is worth noting that, despite all of the dangers associated with walking to school, be it air pollution exposure or traffic accidents, there are also adverse consequences associated with not walking to school, including lower levels of fitness and increased risk of adverse health problems due to a lack of exercise, either now or in the future, such as obesity and diabetes [8], and missed opportunities for educational gains achieved through regular participation in physical activity. For this reason, children should continue to be encouraged to walk to school. By making small changes in the route, including the side of the road travelled along on specific segments, with little effort, some significant reductions in air pollution exposure may be able to be achieved.

## 5. Conclusions

The side of the road on which pedestrians travel can have a significant impact on air pollution exposure on the commute to school. Avoiding the side of the road on which traffic is congested (stop-start conditions) in preference to the free-flow reduces exposure, as would routes consisting of lower traffic flows. Both of these should be taken into account in the design of WSB routes. Adapting the phasing of lights at intersections to minimise wait times for children at school travel times would also help to reduce air pollution exposure amongst school pedestrian commuters. 

## Figures and Tables

**Table 1:** Descriptive statistics of ultrafine particle concentrations. N is the number of 10 s observations, UFP is the ultrafine particle count and STDEV is the standard deviation.

Date	Time	South Walker			North Walker			Car Commuter		
(dd/mm/yy)	(AM/PM)	Mean UFP (cts/cm^3^)	STDEV UFP (cts/cm^3^)	N	Mean UFP (cts/cm^3^)	STDEV UFP (cts/cm^3^)	N	Mean UFP (cts/cm^3^)	STDEV UFP (cts/cm^3^)	N
10/11/2015	AM	14,600	14,000	120	17,500	20,500	120	11,300	12,100	108
11/11/2015	AM	13,400	17,800	120	15,700	17,000	120	3700	1300	78
12/11/2015	AM	25,200	25,000	120	28,500	27,800	120	11,800	2300	84
16/11/2015	AM	12,200	16,800	120	11,400	10,900	120	9000	10,000	54
17/11/2015	AM	23,000	31,700	120	27,400	33,800	120	7300	4300	54
10/11/2015	PM	11,500	11,700	120	11,300	10,100	120	6900	2400	84
11/11/2015	PM	10,700	14,900	120	9700	13,100	120	6800	2600	66
12/11/2015	PM	9900	12,900	120	8500	11,600	120	7800	14,600	84
16/11/2015	PM	7000	9500	120	7400	8900	120	5500	5000	84
19/11/2015	PM	26,200	37,400	120	22,000	24,600	120	17,600	9400	72

## Abbreviations

UFP	Ultrafine particles
WSB	Walking school bus
